# Diagnostic Models for Screening of Periodontitis with Inflammatory Mediators and Microbial Profiles in Saliva

**DOI:** 10.3390/diagnostics10100820

**Published:** 2020-10-14

**Authors:** Jungwon Lee, Jun-Beom Lee, Hyun-Young Song, Min Jung Son, Ling Li, In-Chul Rhyu, Yong-Moo Lee, Ki-Tae Koo, Jung-Sub An, Jin Sup Kim, Eunkyung Kim

**Affiliations:** 1 One-Stop Specialty Center, Seoul National University Dental Hospital, Seoul 03080, Korea; jungwonlee.snudh@gmail.com; 2 Department of Periodontology and Dental Research Institute, School of Dentistry, Seoul National University, Seoul 03080, Korea; dentjblee@gmail.com (J.-B.L.); 1030shy@naver.com (H.-Y.S.); alswhd1030@naver.com (M.J.S.); applemint1228@snu.ac.kr (L.L.); icrhyu@snu.ac.kr (I.-C.R.); ymlee@snu.ac.kr (Y.-M.L.); 3 Department of Orthodontics, Seoul National University Dental Hospital, Seoul 03080, Korea; jungsub.an@gmail.com; 4 R&D Center, Sugentech, Inc., Daejeon 34025, Korea; js.kim@sugentech.com (J.S.K.); ekkim@sugentech.com (E.K.)

**Keywords:** periodontitis, diagnosis, saliva, biomarkers, matrix metalloproteinase, inflammatory mediators

## Abstract

This study aims to investigate and assess salivary biomarkers and microbial profiles as a means of diagnosing periodontitis. A total of 121 subjects were included: 28 periodontally healthy subjects, 24 with Stage I periodontitis, 24 with Stage II, 23 with Stage III, and 22 with Stage IV. Salivary proteins (including active matrix metalloproteinase-8 (MMP-8), pro-MMP-8, total MMP-8, C-reactive protein, secretory immunoglobulin A) and planktonic bacteria (including *Aggregatibacter actinomycetemcomitans*, *Porphyromonas gingivalis*, *Tannerella forsythia*, *Treponema denticola*, *Fusobacterium nucleatum*, *Prevotella intermedia*, *Porphyromonas nigrescens*, *Parvimonas micra*, *Campylobacter rectus*, *Eubacterium nodatum*, *Eikenella corrodens*, *Streptococcus mutans*, *Staphylococcus aureus*, *Enterococcus faecalis*, and *Actinomyces viscosus*) were measured from salivary samples. The performance of the diagnostic models was assessed by receiver operating characteristics (ROCs) and area under the ROC curve (AUC) analysis. The diagnostic models were constructed based on the subjects’ proteins and/or microbial profiles, resulting in two potential diagnosis models that achieved better diagnostic powers, with an AUC value > 0.750 for the diagnosis of Stages II, III, and IV periodontitis (Model PA-I; AUC: 0.796, sensitivity: 0.754, specificity: 0.712) and for the diagnosis of Stages III and IV periodontitis (Model PA-II; AUC: 0.796, sensitivity: 0.756, specificity: 0.868). This study can contribute to screening for periodontitis based on salivary biomarkers.

## 1. Introduction

The diagnosis of periodontitis is conventionally based on clinical evaluation, including probing pocket depth, bleeding on probing, clinical attachment level, periodontal index and gingival index, and radiographic examinations. However, the conventional method has a limitation in that some parameters reflect only past evidence of inflammatory changes and do not show that this inflammatory change would progress or regress in the future [1]. To overcome the aforementioned limitation, various tools for the detection of periodontitis have been developed and introduced [2,3].

The use of saliva for oral-based diagnostics has proven to be easy to use for point of care (POC) application. Oral-based diagnostics have been developed to detect several pathologies, including oral cancer, human immunodeficiency virus infection, hepatitis C infection, and Ebola virus infection, with the advantages of being readily accessible and minimally invasive [4,5,6,7]. The ease of access and sampling of saliva containing inflammatory cytokines and microbial or viral infection provides potential possibilities for its use in the diagnosis of periodontitis [8].

It has been observed that there is an equilibrium in the interaction between the host and microorganisms when the periodontal apparatus is healthy [9]. This ecosystem can be affected by genetic factors of the host and environmental factors, including smoking [10]. The development of periodontal pathology is the outcome of several changes in the host or environmental state, indicating a possible relationship between the risk of periodontitis and susceptibility factors [11].

A previous study has demonstrated that microbial variables could show a discriminating potential in distinguishing subjects with periodontitis [3]. Dysbiosis of the oral microbiome can induce the immune reaction of the host, resulting in the release of various inflammatory mediators, including matrix metalloproteinase-8 (MMP-8), C-reactive protein (CRP), and secretory IgA (sIgA) [12,13,14].

MMP-8 (also known as type neutrophil collagenase or collagenase 2) has substrate specificity for type I collagen, accounting for periodontal extracellular matrix; thus, attention has been paid to MMP-8 in periodontitis [15,16]. Human MMP-8 is produced and secreted as a 55~80 kDa glycosylated inactive precursor form and is activated when cleaved by extracellular proteinases, resulting in the removal of the prodomain [17]. Inactive proMMP-8 can be activated by the “cystein switch” depending on several factors, including bacterial proteinase, temperature, pH value, calcium ion, and oxygen radicals [18]. Various studies have demonstrated that active MMP-8(aMMP-8) is related to periodontal pathogenesis [19,20,21]. Meanwhile, it is expected that the amount of aMMP-8 will also appear high in individuals with high expression of MMP-8. Therefore, it is necessary to measure all forms of MMP-8 (proMMP-8, aMMP-8, and total MMP-8) to examine the relationship between MMP-8 and the severity of periodontitis.

A variety of these microbial profiles and inflammatory mediators in saliva have been investigated as diagnostic tools for the detection of periodontitis. Although this diagnostic method has shown potential for identifying periodontitis, some limitations have been reported for this diagnostic tool, which uses a single biomarker [8]. There was a study that showed an improvement of sensitivity and specificity in detection using a model that combined related biomarkers [22]. As periodontitis is a multifactorial disease, a model combining microbial profiles and inflammatory mediators involved in chronic infection might improve its diagnostic accuracy.

Recently, for the purpose of reflecting the severity, extent, and complexity of periodontal breakdown, a new classification of periodontitis has been suggested [23]: Stage I, initial periodontitis; Stage II, moderate periodontitis; Stage III, severe periodontitis with potential for additional tooth loss; Stage IV, advanced periodontitis with extensive tooth loss and potential for loss of dentition. However, little evidence has been accumulated in support of using salivary biomarkers for such a classification system.

The aim of this study is to verify alterations in salivary biomarkers, including microbial profiles and inflammatory mediators, according to periodontal status and to investigate a combined model for periodontitis in accordance with the new classification.

## 2. Materials and Methods

### 2.1. Subjects

This clinical study was approved by the Institutional Review Board of Seoul National University Dental Hospital (CRI19004), approved on 4 April 2019. The subjects in this study were recruited from July 2019 to December 2019. Upon receiving written consent, 121 human subjects, aged 18 years or older, were evaluated at the Department of Periodontology in the Seoul National University Dental Hospital. All subjects involved in this study were required to have 20 or more teeth. In addition, the exclusion criteria applied was as follows: (a) use of any antibiotics or anti-inflammatory drugs within 3 months of registration; (b) use of an immunosuppressant (methotrexate, leflunomide, tacrolimus, cyclosporin, azathioprine) or adrenal cortical hormone (oral or injection) within 3 months of registration; (c) having less than 20 teeth; (d) having uncontrolled hypertension or diabetes; (e) subjects who have serious cardiovascular disease, respiratory system disease, kidney disease, liver disease, digestive system disease, blood system disease, or neuropsychiatric disease; (f) subjects with hyperthyroidism or hypothyroidism; (g) women who were pregnant or planning to become pregnant; (h) subjects with autoimmune diseases; (i) subjects with a history or presence of malignant tumors in the jawbone; (j) subjects who have had a history of or are currently using drugs or alcohol abuse within one year; (k) subjects with other inflammatory diseases in the oral cavity besides periodontitis, such as stomatitis (including ulcerative, blistering, erosive) or oral cancer; (l) subjects with other inflammatory diseases in the oral cavity besides periodontitis, such as ulcers, simple herpes and shingles, or fungal or bacterial infections; (m) subjects whose participation was judged by the researcher to be inappropriate because their involvement may cause ethical problems or seriously affect the research results.

### 2.2. Clinical and Radiographic Examination

A consent form was signed by and obtained from each subject following a sufficient explanation of the study. To identify if the subject was suitable for this study, demographic information such as gender and date of birth was obtained, and the systemic conditions of the participants were also examined.

On the first visit, clinical examinations were performed, and the following parameters were recorded. The gingival index (GI) [24] and plaque index (PI) [25] were examined on the buccal and lingual surfaces of the teeth. Additionally, the probing pocket depth (PPD), gingival recession (GR), and clinical attachment level (CAL) were measured at the six sites around the tooth. The amount of tooth loss (TL), sites of bleeding on probing (BOP), tooth mobility (TM), and furcation involvement (FI) were recorded per tooth. Alveolar bone loss at the mesial and distal site of the tooth was measured with periapical radiographs. The subjects were classified as healthy or Stages I, II, III, or IV depending on the severity, extent, and complexity of their periodontitis [23].

### 2.3. Preparation of Solution for Saliva Storage

Approximately 0.1 M phenylmethylsulphonyl fluoride (PMSF) stock solution, dissolved in isopropanol, was stored at room temperature and 0.5 M ethylenediaminetetraacetic acid (EDTA) stock solution, dissolved in distilled water (DW), was refrigerated. The two stock solutions were stored independently due to the instability of PMSF when mixed with 1X phosphate-buffered saline (PBS).

### 2.4. Whole Saliva with Draining Method

Unstimulated saliva was collected with the participant’s head tilted slightly forward in a sitting position by drooling into a funnel-shaped test tube. The sampling was performed for 15 min and was stopped when the amount collected reached 5 mL. Subsequently, the saliva sample was placed on ice and supplemented with 1X PBS 4930 μL, 20 μL EDTA solution, and 50 μL PMSF stock solution; then, vortexing was performed. The samples were stored in a deep freezer at a temperature of −80 °C immediately after collection for the preservation of biomarkers.

### 2.5. Saliva Collection for Oral Microbial Identification

After collecting GCF, subjects took another break for 5 min. The subjects gargled and rinsed with gargle solution (EasyperiO kit, YD Life Science Company, Gyeonggi-do, Korea) for 30 s and then spit it into the sample container. The sample container cap was closed tightly. Samples were stored in a refrigerator (4 °C) and transported to the analytical company (YD Life Science company, Gyeonggi-do, Korea) on dry ice.

### 2.6. Protein Biomarker Assays

Protein biomarker levels were detected with enzyme-linked immunosorbent assays (ELISAs) for measurement of active MMP-8 (Human MMP-8 activity assay, QuickZyme Biosciences B.V., Leiden, The Netherlands), total MMP-8 (Human MMP-8 ELISA Kit, RayBiotech, Inc., Norcross, GA, USA), CRP (Salivary C-Reactive Protein ELISA Kit, Salimetrics, LLC, State College, PA, USA), and sIgA (Secretory IgA ELISA Kit, Demeditec Diagnostics GmbH, Kiel, Germany), according to the manufacturers’ protocols. The total MMP-8 ELISA Kit is a sandwich method, and the active MMP-8 ELISA Kit is a method of indirectly measuring the amount of active MMP-8 by measuring the chromogenic substrate that is cleaved by the active detection enzyme activated by the active MMP-8. Pro-MMP-8 was calculated by subtracting active MMP-8 from total MMP-8. Pure recombinant human MMP-8 protein (aa 21–467; Sino Biological, Cat. No. 10254-HNAH, Beijing, China) was used as a positive control. Measurements were performed in duplicate to ensure repeatability of the experimental analysis.

### 2.7. Detection of Microbial Profiles

The samples were analyzed by quantitative analysis using multiplex-quantity real-time polymerase chain reaction (PCR) according to the manufacturer’s protocol (EasyPerio; YD Life Science, Gyeonggi-do, Korea) for 15 oral pathogenic bacteria: *Aggregatibacter actinomycetemcomitans* (*A. actinomycetemcomitans*, *Aa*), *Porphyromonas gingivalis* (*P. gingivalis*, *Pg*), *Tannerella forsythia* (*T. forsythia*, *Tf*), *Treponema denticola* (*T. denticola*, *Td*), *Fusobacterium nucleatum* (*F. nucleatum*, *Fn*), *Prevotella intermedia* (*P. intermedia*, *Pi*), *Porphyromonas nigrescens* (*P. nigrescens*, *Pn*), *Parvimonas micra* (*P. micra*, *Pm*), *Campylobacter rectus* (*C. rectus*, *Cr*), *Eubacterium nodatum* (*E. nodatum*, *En*), *Eikenella corrodens* (*E. corrodens*, *Ec*), *Streptococcus mutans* (*S. mutans*, *Smu*), *Staphylococcus aureus* (*S. aureus*, *Sa*), *Enterococcus faecalis* (*E. Faecalis*, *Ef*), and *Actinomyces viscosus* (*A. viscosus*, *Av*). Patient-based microbial data was analyzed. Sequences of the primers and probes used for real-time PCR are shown in Table 1. To obtain a standard curve for each target, plasmid DNA of each strain at a concentration of 10^4^ copies/mL was prepared, and PCR was performed. The copy value for each strain was calculated by substituting the Ct value of each bacteria into a standard curve. Measurements were performed in duplicate to ensure repeatability of the experimental analysis.

### 2.8. Sample Size Calculation

The sample size was established according to the previous study by Mauramo M et al. [26], reporting the diagnostic performance of MMP-8 for periodontitis with an AUC value of 0.67. With a 95% confidence interval and 80% power, the sample size was calculated as a total of 108 subjects [27]. Give a 10% drop-out rate, 30 subjects were to be collected for each group of periodontitis.

### 2.9. Statistical Analysis

Continuous data were represented with means and standard deviation for each subject group. Group comparisons were made using one-way ANOVA in SPSS version 17 (IBM Software, Armonk, NY, USA). Dichotomized data were represented with a number and percentage for each subject group. Group comparisons were made with Fisher’s exact tests. Differences were considered statistically significant when *p*-values < 0.05. Seven diagnostic models of periodontitis (Model PD), namely, Stage I periodontitis (Model PD-I), Stage II periodontitis (Model PD-II), Stage III periodontitis (Model PD-III), Stage IV periodontitis (Model PD-IV), periodontitis above Stage I (Stages II, III, and IV; Model PA-I), and periodontitis above Stage II (Stages III and IV; Model PA-II), were constructed according to the concentration of proteins and microbial profiles based on a forward stepwise logistic regression analysis using SPSS statistics software (version 21.0, IBM Software, Armonk, NY, USA). The diagnostic models were evaluated by sensitivity, specificity, and ROC curve analysis using Excel (Microsoft 365, Redmond, WA, USA).

## 3. Results

### 3.1. Demographic and Clinical Characteristics of Subjects

**Figure 1:**
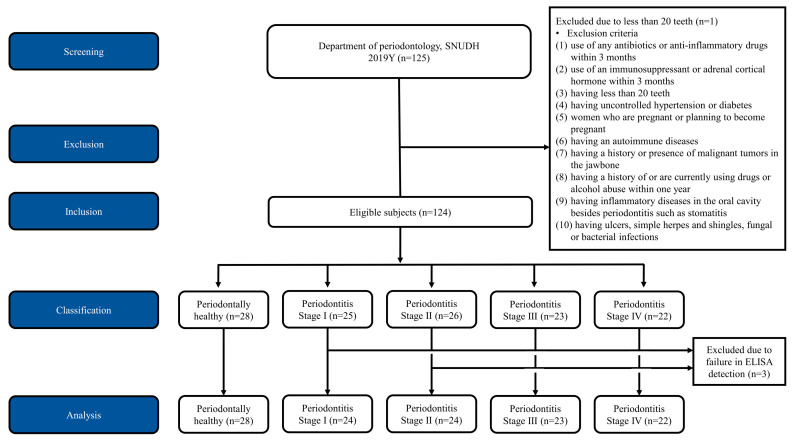
Consolidated standards of reporting trials (CONSORT) flow diagram of the study. 125 subjects were screened and one subject was excluded due to lack of teeth. Finally 124 subjects were included in this study and allocated into the five groups of periodontal status including healthy, periodontitis stage I, II, III, and IV. Three subjects were excluded in the analysis due to failure in enzyme-linked immunosorbent assay (ELISA) dectection. A total 121 subjects were included in the final analysis.

Thirty-eight male (30.4%) and eighty-seven female (69.6%) subjects, ranging in age from 20 to 79 years, were enrolled in the study. Following the recording of clinical and radiographic parameters, the subjects were allocated into the five groups of periodontal status. One subject was excluded due to the lack of teeth. The data of three subjects were excluded because the proteins in their whole-saliva samples, including active MMP-8, pro-MMP-8, and total MMP-8, did not show detectable levels. A total of 121 subjects were included in the final analysis (Figure 1). Analysis of the data obtained from the healthy (*n* = 28) and periodontitis populations (*n* = 93; Stage I: 24, Stage II: 24, Stage III: 23, and Stage IV: 22) was performed in this study.

The demographic data (Table 2) for systemic disease, including osteoporosis and hepatitis, were balanced among the five groups. However, age, sex, hypertension, diabetes mellitus, and smoking were statistically and significantly different among the groups. The subjects with hypertension, diabetes, and smoking were not found in the periodontally healthy group; however, they were significantly associated with Stage III and Stage IV periodontitis. In addition, the older participants were significantly found to have higher stages of periodontitis.

Dental and periodontal data (Table 2) were significantly different among the five groups for mean GR (0.11 to 0.65; *p* < 0.001), mean PPD (2.36 to 2.83 mm; *p* = 0.003), mean CAL (0.24 to 1.25 mm; *p* < 0.001), mean MBL (0.077 to 2.34 mm; *p* < 0.001), number of furcation-involved teeth (0 to 0.73; *p* < 0.001), number of immobile teeth (24.09 to 27.25; *p* < 0.001), number of mobile teeth with Grade 1 (0 to 1.64; *p* < 0.001), number of mobile teeth with Grade 2 (0 to 0.27; *p* = 0.001), and number of mobile teeth with Grade 3 (0 to 0.23; *p* = 0.022).

### 3.2. Inflammatory Mediators and Microbial Profiles in Saliva

**Figure 2:**
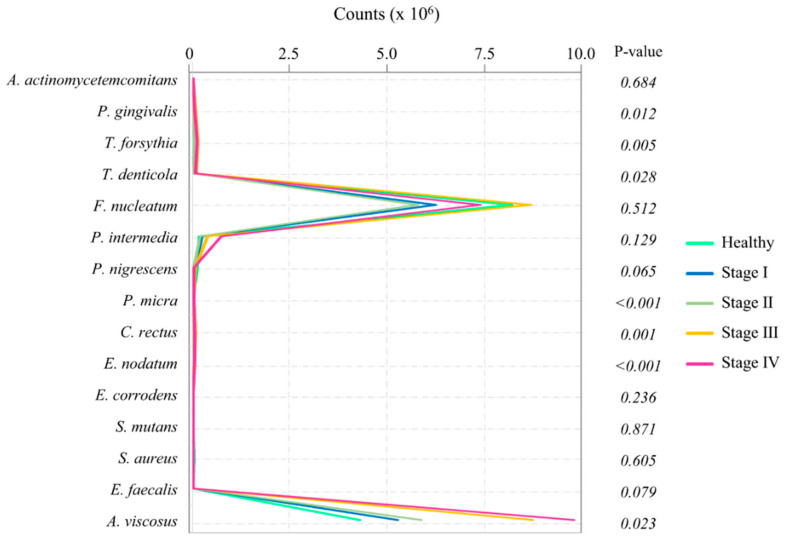
Changes in profiles of salivary bacteria according to stages of periodontitis (×10^6^).

The data of proteins are shown in Table 2. The levels of protein concentrations of active MMP-8 (*p* < 0.001), pro-MMP-8 (*p* < 0.001), total MMP-8 (*p* < 0.001), and sIgA (*p* = 0.012) showed significant differences among the groups. The overall microbial profiles are presented in Figure 2. The level of *P. gingivalis* (*p* = 0.012), *T. forsythia* (*p* = 0.005), *T. denticola* (*p* = 0.028), *P. micra* (*p* < 0.001), *C. rectus* (*p* = 0.001), *E. nodatum* (*p* < 0.001), and *A. viscosus* (*p* = 0.023) showed significant differences among the groups.

### 3.3. Construction of Diagnostic Models Based on Inflammatory Mediators in Saliva

Diagnostic models of periodontitis (Model PD), Stage I periodontitis (Model PD-I), Stage II periodontitis (Model PD-II), Stage III periodontitis (Model PD-III), Stage IV periodontitis (Model PD-IV), periodontitis above Stage I (Stages II, III, and IV; Model PA-I), and periodontitis above Stage II (Stages III, and IV; Model PA-II) were constructed using the proteins and microbial profiles that showed significant differences.

The Model PD mathematical formula, with an accuracy of 0.802 (Table 3), was constructed to discriminate the periodontitis groups from the healthy group using logistic regression analysis (Equation (1)). This model showed high sensitivity (1.000), but low specificity (0.143) (Table 3).

(1) $$\mathrm{Model}\;\mathrm{PD}=\frac{1}{{1\;+\;e}^{-(-0.066\;+\;0.002\;\times \;T-\mathrm{MMP}8\;+\;0.505\;\times \;\mathrm{En})}}$$

The Model PD-I mathematical formula, with an accuracy of 0.785 (Table 3), was constructed to diagnose the Stage I periodontitis group from the healthy group and Stage II, III, and IV periodontitis patients using logistic regression analysis (Equation (2)). This model showed high specificity (0.969) but low sensitivity (0.042) (Table 3).

(2) $$\mathrm{Model}\;\text{PD-I}=\frac{1}{{1\;+\;e}^{-(-0.825\;-\;10.113\;\times \;\text{Active-MMP}8\;-\;10.125\;\times \;\text{Pro-MMP}8\;+\;10.117\;\times \;\text{Total-MMP}8)}}$$

The Model PD-II mathematical formula, with an accuracy of 0.760 (Table 3), was constructed to diagnose the Stage II periodontitis group from the healthy group and Stage I, III, and IV periodontitis patients using logistic regression analysis (Equation (3)). This model showed high specificity (0.948) but low sensitivity (0.000) (Table 3).

(3) $$\mathrm{Model}\;\text{PD-II}=\frac{1}{{1\;+\;e}^{-(-1.308\;-\;0.513\;\times \;\mathrm{Pm}\;+\;0.293\;\times \;\mathrm{Cr})}}$$

The Model PD-III mathematical formula, with an accuracy of 0.835 (Table 3), was constructed to diagnose the Stage III periodontitis group from the healthy group and Stage I, II, and IV periodontitis patients using logistic regression analysis (Equation (4)). This model showed high specificity (0.959) but low sensitivity (0.304) (Table 3).

(4) $$\mathrm{Model}\;\text{PD-III}=\frac{1}{{1\;+\;e}^{-(-2.946\;+\;10.998\;\times \;\text{Active-MMP}8\;+\;10.999\;\times \;\text{Pro-MMP}8\;-\;10.999\;\times \;\text{Total-MMP}8\;-\;0.259\;\times \;\mathrm{Td}\;+\;0.419\;\times \;\mathrm{Pm}\;+\;0.419\;\times \;\mathrm{En})}}$$

The Model PD-IV mathematical formula, with an accuracy of 0.860 (Table 3), was constructed to diagnose the Stage IV periodontitis group from the healthy group and Stage I, II, and III periodontitis patients using logistic regression analysis (Equation (5)). This model showed high specificity (0.980) but low sensitivity (0.318) (Table 3).

(5) $$\mathrm{Model}\;\text{PD-IV}=\frac{1}{{1\;+\;e}^{-(-12.023\;+\;0.004\;\times \;\text{Pro-MMP}8\;+\;0.427\;\times \;\mathrm{En}\;+\;1.267\;\times \;\mathrm{Av})}}$$

The Model PA-I mathematical formula, with an accuracy of 0.736 (Table 3), was constructed to discriminate the Stage II, III, and IV periodontitis groups from the healthy group and Stage I periodontitis patients using logistic regression analysis (Equation (6)). This model showed improved sensitivity (0.754) and specificity (0.712) (Table 3).

(6) $$\mathrm{Model}\;\text{PA-I}=\frac{1}{{1\;+\;e}^{-(-1.635\;+\;0.005\;\times \;\text{Pro-MMP}8\;+\;0.193\;\times \;\mathrm{Cr}\;+\;0.322\;\times \;\mathrm{En})}}$$

The Model PA-II mathematical formula, with an accuracy of 0.826 (Table 3), was constructed to discriminate the Stage III and IV periodontitis groups from the healthy group and Stage I and II periodontitis patients using logistic regression analysis (Equation (7)). This model showed good specificity (0.756) and sensitivity (0.868) (Table 3).

(7) $$\mathrm{Model}\;\text{PA-II}=\frac{1}{{1\;+\;e}^{-(-11.310\;+\;0.003\;\times \;\mathrm{sIgA}\;+\;0.300\;\times \;\mathrm{Pg}\;-\;0.302\;\times \;\mathrm{Tf}\;+\;0.324\;\times \;\mathrm{Pm}\;+\;0.675\;\times \;\mathrm{En}\;+\;1.231\;\times \;\mathrm{Av})}}$$

### 3.4. Validation of Multianalyte Models

**Figure 3:**
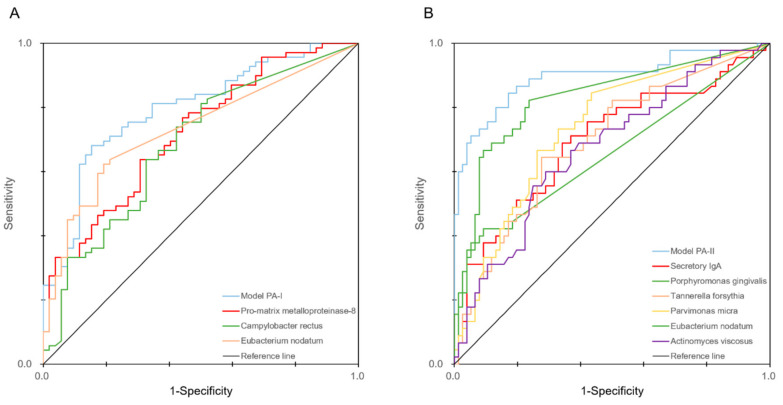
ROC curves of the PA-I (**A**) and PA-II (**B**) models. The ROC curves of the biomarkers that were significantly related and included in each model are also included in each figure.

Among the seven models, Model PA-I and Model PA-II, which showed good sensitivity and specificity, were further investigated with the ROC curves (Figure 3).

It was found that the models combining salivary biomarkers and microbial profiles were useful for discriminating periodontal status. The AUC values of pro-MMP-8, *C. rectus*, and *E. nodatum* were 0.720, 0.685, and 0.733, respectively. In contrast, the AUC value of Model PA-I, which combined the markers, was 0.796. The AUC values of sIgA, *P. gingivalis*, *T. forsythia*, *P. micra*, *E. nodatum*, and *A. viscosus* were 0.695, 0.653, 0.694, 0.736, 0.831, and 0.673, respectively, while Model PA-II, which combined them, showed an AUC value of 0.894.

## 4. Discussion

In this study, we built the seven diagnostic models for periodontitis by combining meaningful salivary biomarkers (including inflammatory mediators and microbial profiles) that significantly changed with the stages of periodontitis. Among the seven models, PA-I and PA-II were both highly sensitive and specific compared with PD-I, -II, -III, and -IV models. In Model PA-I, *E. nodatum* and *C. rectus* were included; these bacteria belong to the orange complex, which has a role in linking early colonizing and pathogenic bacteria of periodontitis. Therefore, it is thought that these bridging species are very important in the diagnosis of periodontitis with Stages II, III, and IV of the disease. Meanwhile, in Model PA-II, the purple complex (such as *A. viscosus*), orange complex (such as *P. micra* and *E. nodatum*), and red complex (such as *P. gingivalis* and *T. forsythia*) were included. The high accuracy of Model PA-II means that overall planktonic bacterial species can increase when the severity of periodontitis increases because various species consisting of early colonizer, bridging species, and pathogenic bacteria are included. Simple counting of bacteria also showed that there were more groups of streptococcus and *A. viscosus* in Stage IV, meaning the more severe the stage of periodontitis is, the more overall bacteria there are.

To our knowledge, there is little literature to validate a new classification system of periodontitis in the development of a diagnostic method, including salivary biomarkers. In 2017, the new classification system for case definitions of periodontitis was developed and suggested based on cumulative studies for almost 20 years [28]. In the workshop, three obviously different forms of periodontitis based on pathophysiology were categorized: necrotizing periodontitis, periodontitis as a direct manifestation of systemic disease, and periodontitis. Herein, we included the latter form. The periodontitis stage increased according to the severity, complexity, extent, and distribution of the disease. Our prediction models for periodontitis showed high accuracy based on this new classification system by using limited amounts of significant biomarkers.

In this study, we included both pro- and active forms of MMP-8. Several studies found that the active forms of MMP could distinguish periodontitis [19,29,30,31,32,33,34]; however, in this study, two forms were significantly different among all stages of periodontitis. The increase was considered to reflect the increased leakage of all types of MMP-8 according to the severity of periodontitis. Interestingly, CRP did not show a significant difference, which was not consistent with other studies [35,36]. This might be because the detective capacity of ELISA is not sensitive enough to detect the changes in the level of CRP [37,38]. Therefore, to monitor the level of CRP, more sensitive techniques should be used.

Contrary to our expectations, all periodontopathogenic bacteria were not detected in the saliva in periodontitis patients. This could be a result of the fact that almost all the patients showed periodontitis in a “localized” form, and the amount of planktonic bacteria was too low to be detected. Further studies should be performed in patients with “generalized” periodontitis.

SIgA indicates adaptive immunity and is widely used for a diagnosis of periodontitis. Mesa et al. reported similar results showing sIgA was not significantly different between healthy patients and periodontitis groups [39]. Chronic inflammation increased cortisol levels and, inversely, decreased sIgA [40]. In the present study, sIgA seemed to show higher levels in Stages III and IV of periodontitis compared with other groups, but there were no statistical differences.

In spite of the limitations of the cross-sectional study, our findings suggest that whole saliva might be used as a diagnostic tool for periodontitis. Additionally, the proper selection of biomarkers in whole saliva is important in order to increase the sensitivity and specificity of the diagnosis of periodontitis [41,42]. On the other hand, this method had limitations in the early diagnosis of Stage I. Other studies also showed similar limitations, where the screening test could not distinguish early-stage periodontitis [43]. By definition, Stage I periodontitis is a bridge between gingivitis and periodontitis; thus, it can show lower levels of biomarkers than the severe forms. Other researchers have tried to discriminate gingivitis and periodontitis through macrophage inflammatory protein-1α [44,45]. To overcome the problem, it is necessary to find additional biomarkers or develop more sensitive techniques for early detection.

The diagnosis of periodontitis with clinical parameters is a very effective tool, but it is time-consuming and labor-intensive. The evaluation of clinical parameters is somewhat difficult to standardize and cannot monitor the real-time changes in periodontal disease progression [41,42]. Therefore, the method of whole-saliva analysis could be an easier and simpler diagnostic tool for the detection of periodontitis. Additionally, salivary biomarkers can be a very prospective screening tool when one considers the high correlations between periodontitis and systemic disease [29,46,47].

## 5. Conclusions

This study can contribute to screening for periodontitis based on salivary biomarkers. Two potential diagnosis models, PA-I for the diagnosis of Stage II, III, and IV periodontitis and PA-II for the diagnosis of Stage III and IV periodontitis, showed the highest performance with biomarkers in whole saliva.

## Figures and Tables

**Table 1:** Sequence of primers/probes used in PCR.

	Target Gene	Accession No.	Primer and Probe	Sequence (5′–>3′)	Length	Size (bp)
*Aggregatibacter* *actinomycetemcomitans*	16S ribosomal RNA	M75039.1	Forward primer	CAAGTGTGATTAGGTAGTTGGTGGG	25	220
			Reverse primer	CCTTCCTCATCACCGAAAGAA	21	
			Probe	ATCGCTAGCTGGTCTGAGAGGATGGCC	27	
*Porphyromonas* *gingivalis*	FimA type II (fimA)	KF770042.1	Forward primer	GGAGTCTAATCTATTCGGTGCTTC	24	132
			Reverse primer	GTGTAGTCTCTTCCCAACCAG	21	
			Probe	ATTTCAACGGTGCTTATTCCCCTGC	25	
*Tannerella* *forsythia*	karilysin protease gene	GQ856797	Forward primer	TGGAGAATCAGTAACGGTTGG	21	149
			Reverse primer	CCCCAACCACATTCACTACG	20	
			Probe	TCCATTAAGCCCATTGCCCGGAA	23	
*Treponema* *denticola*	OpdB gene	AF355459	Forward primer	TCCGAGTGTTTACAGCCTTG	20	94
			Reverse primer	GTCCTCATACCACTTTTCTCCC	22	
			Probe	CTTCCGCCCCTGATTTGAGCAAC	23	
*Fusobacterium* *nucleatum*	16S ribosomal RNA gene	FJ471640	Forward primer	CGATAAGTAATCCGCCTGGG	20	137
			Reverse primer	TCCTAAGATGTCAAACGCTGG	21	
			Probe	CGTCGAATTAAACCACATGCTCCACC	26	
*Prevotella* *intermedia*	hemagglutinin (phg) gene	AF017417	Forward primer	ACATTGGAACTGAGACACGG	20	145
			Reverse primer	GCCTCACTTTACTCCCCAAC	20	
			Probe	TCAATCCTGCACGCTACTTGGCT	25	
*Prevotella* *nigrescens*	gyrase subunit B (gyrB) gene	KF186729	Forward primer	AGAATACGAACAGGGAAAGCC	21	136
			Reverse primer	ACGTGCAACTATATCCCACTG	21	
			Probe	TTGGTGAAACCGATAAGACTGGTACGC	29	
*Parvimonas* *micra*	16S ribosomal RNA gene	AF542231	Forward primer	ATGAATGCTAGGTGTTGGGAG	21	141
			Reverse primer	GAATTAAACCACATGCTCCGC	23	
			Probe	AGTTTCAGTGTTGCCACCGTACTCC	25	
*Campylobacter* *rectus*	groEL gene	AB071388	Forward primer	GCTACGGAGACTGAGATGAAAG	22	136
			Reverse primer	TTAGATCCACTTTCGCACCG	20	
			Probe	ATGCCTTCTTCTACAGCCGCCTT	23	
*Eubacterium* *nodatum*	16S ribosomal RNA gene	U13041.1	Forward primer	TCGTAAACTTCTGTCCAAAGGG	22	138
			Reverse primer	CACCTACATACTCTTTACGCCC	22	
			Probe	TAATTCCGGATAACGCTCGCCCC	23	
*Eikenella* *corrodens*	proline iminopeptidase (pip)	AY198131	Forward primer	CCTAACGATATGCCTGGAACC	21	149
			Reverse primer	TCGATACTCCGTTTGCCATC	20	
			Probe	AGTTTGCCGCCTAGTTTCATCCCT	24	
*Streptococcus* *mutans*	PTS EII (mtlA)	AF210133	Forward primer	CCTTCCTAGTCGCTTCCATTATC	23	112
			Reverse primer	ACTGCTTGACCTTTAGACTCG	23	
			Probe	TTTTGCTGCTTGTGTCACTGCTTGT	27	
*Staphylococcus* *aureus*	translation elongation factor Tu (tuf) gene	AF298796.1	Forward primer	CTTCCCAGGTGACGATGTAC	22	132
			Reverse primer	TCACGTTCTGGAGTTGGAATG	23	
			Probe	AGCTTTAGAAGGCGATGCTCAATACGAA	28	
*Enterococcus* *faecalis*	16S ribosomal RNA gene	EU887827.1	Forward primer	AACTGTTCATCCCTTGACGG	22	137
			Reverse primer	TCAGACTTAAGAAACCGCCTG	21	
			Probe	ACGCTTGCCACCTACGTATTACCG	24	
*Actinomyces* *viscosus*	16S rRNA gene	X82453.1	Forward primer	GGGAGCGAACAGGATTAGATAC	24	148
			Reverse primer	CCTTTGAGTTTTAGCCTTGCG	23	
			Probe	ACACCTAGTGCCCAACGTTTACGG	24	
GAPDH	Human ORF	LT737735.1	Forward primer	GAAGGTGAAGGTCGGAGTC	19	220
			Reverse primer	GAAGATGGTGATGGGATTTC	20	
			Probe	CAAGCTTCCCGTTCTCAGCC	20	
beta-actin	Homo sapiens actin beta (ACTB), mRNA	NM_001101.4	Forward primer	AGTCCCTTGCCATCCTAAAAG	22	92
			Reverse primer	CAATGCTATCACCTCCCCTG	21	
			Probe	CCAGTCCTCTCCCAAGTCCACAC	24	

**Table 2:** Demographic characteristics, clinical periodontal parameters, salivary biomarkers, and planktonic bacteria of each group (mean ± standard deviation).

	Healthy Controls (HC)	Periodontitis (PD)				Significance
		PS-I	PS-II	PS-III	PS-IV	
	(*n* = 28)	(*n* = 28)	(*n* = 28)	(*n* = 28)	(*n* = 28)	
Age	30.04 ± 8.79	35.00 ± 15.10	49.21 ± 16.92	58.17 ± 14.40	61.41 ± 11.35	<0.001
Sex						
Male	4 (14.3%)	3 (12.5%)	11 (45.8%)	9 (39.1%)	10 (45.5%)	0.010
Female	24 (85.7%)	21 (87.5%)	13 (54.2%)	14 (60.9%)	12 (54.5%)	
Hypertension	0	1 (4.2%)	1 (4.2%)	4 (17.4%)	6 (27.3%)	0.005
Diabetes	0 (0.0%)	1 (4.2%)	0 (0.0%)	2 (8.7%)	4 (18.2%)	0.023
Osteoporosis	0 (0.0%)	1 (4.2%)	0 (0.0%)	0 (0.0%)	0 (0.0%)	0.769
Hepatitis	0 (0.0%)	0 (0.0%)	1 (4.2%)	0 (0.0%)	0 (0.0%)	0.769
Smoking	0 (0.0%)	0 (0.0%)	2 (8.3%)	4 (17.4%)	1 (4.5%)	0.032
# of teeth	27.68 ± 1.467	27.44 ± 1.227	27.46 ± 1.560	27.56 ± 1.635	26.59 ± 1.681	0.12
BOP, %	30 ± 18	37 ± 19	28 ± 20	35 ± 23	32 ± 24	0.595
Mean GI	0.58 ± 0.57	0.66 ± 0.45	0.50 ± 0.39	0.66 ± 0.69	0.75 ± 0.55	0.592
Mean PI	0.48 ± 0.41	0.51 ± 0.45	0.42 ± 0.45	0.73 ± 0.58	0.61 ± 0.51	0.184
Mean PPD	2.36 ± 0.26	2.54 ± 0.30	2.54 ± 0.33	2.70 ± 0.56	2.83 ± 0.62	0.003
Mean GR	0.11 ± 0.33	0.053 ± 0.056	0.12 ± 0.15	0.26 ± 0.27	0.65 ± 0.97	<0.001
Mean CAL	0.24 ± 0.58	0.12 ± 0.12	0.27 ± 0.29	0.57 ± 0.59	1.25 ± 1.43	<0.001
Mean ABL	0.077 ± 0.32	0.22 ± 0.16	0.50 ± 0.27	1.18 ± 0.71	2.34 ± 2.07	<0.001
# of FI	0	0	0	0.36 ± 0.78	0.73 ± 1.32	<0.001
TM0	27.25 ± 2.45	27.20 ± 1.35	27.29 ± 1.33	26.36 ± 1.98	24.09 ± 3.31	<0.001
TM1	0	0	0	0.80 ± 1.38	1.64 ± 2.08	<0.001
TM2	0	0	0	0	0.27 ± 0.63	0.001
TM3	0	0	0	0.04 ± 0.20	0.23 ± 0.61	0.022
Sal. Ez.						
Active MMP-8 (ng/mL)	119.10 ± 93.73	172.82 ± 166.54	190.57 ± 125.68	281.87 ± 206.93	288.35 ± 145.54	<0.001
Pro-MMP-8 (ng/mL)	115.90 ± 90.62	127.38 ± 82.44	155.02 ± 93.70	217.72 ± 104.60	256.90 ± 139.43	<0.001
Total MMP-8 (ng/mL)	235.01 ± 178.71	300.21 ± 233.94	345.59 ± 209.98	499.57 ± 286.48	545.24 ± 270.87	<0.001
CRP (ng/mL)	0.93 ± 3.01	0.82 ± 1.68	0.47 ± 1.18	0.70 ± 1.10	0.37 ± 0.80	0.564
sIgA (μg/mL)	225.16 ± 135.68	221.45 ± 133.02	221.25 ± 115.93	321.20 ± 163.72	336.21 ± 184.33	0.012

Abbreviations: PS-I: Stage I periodontitis; PS-II: Stage II periodontitis; PS-III: Stage III periodontitis; PS-IV: Stage IV periodontitis; BOP: bleeding on probing; GI: gingival inflammation; PI: plaque index; PPD: probing pocket depth; GR: gingival recession; CAL: clinical attachment loss; ABL: alveolar bone loss; FI: furcation-involved tooth; TM0: immobile tooth with 0°; TM1: mobile tooth with 1°; TM2: mobile tooth with 2°; TM3: mobile tooth with 3°; Sal. Ez.: salivary enzyme; MMP: matrix metalloproteinase; CRP: C-reactive protein; sIgA: secretory IgA.

**Table 3:** The sensitivity, specificity, and accuracy of each prediction model.

Total	Clinical Final Diagnosis		Prediction Model		FP	FN	Sensitivity		Specificity		Accuracy	
121	HC	28										
	Periodontitis	93	PD	117	24	0	1.000	(93/93)	0.143	(4/28)	0.802	(97/121)
	PS-I	24	PD-I	4	3	23	0.042	(1/24)	0.969	(94/97)	0.785	(95/121)
	PS-II	24	PD-II	5	5	24	0.000	(0/24)	0.948	(92/97)	0.760	(92/121)
	PS-III	23	PD-III	11	4	16	0.304	(7/23)	0.959	(94/98)	0.835	(101/121)
	PS-IV	22	PD-IV	9	2	15	0.318	(7/22)	0.980	(97/99)	0.860	(104/121)
	PS-II, -III, and -IV	69	PA-I	67	15	17	0.754	(52/69)	0.712	(37/52)	0.736	(89/121)
	PS-III and -IV	45	PA-II	44	10	11	0.756	(34/45)	0.868	(66/76)	0.826	(100/121)

Abbreviations: HC: healthy controls; PS-I: Stage I periodontitis; PS-II: Stage II periodontitis; PS-III: Stage III periodontitis; PS-IV: Stage IV periodontitis; FP: false-positive; FN: false-negative.
